# With a Little Help from Their Peers: The Impact of Classmates on Adolescents’ Development of Prosocial Behavior

**DOI:** 10.1007/s10964-020-01260-8

**Published:** 2020-06-11

**Authors:** Robert Busching, Barbara Krahé

**Affiliations:** University of Potsdam, Potsdam, Germany

**Keywords:** Prosocial behavior, Adolescence, Development, Gender, Longitudinal, Multilevel

## Abstract

Peer groups are critical socialization agents for the development of social behavior in adolescence, but studies examining peer-group effects on individuals’ prosocial behavior are scarce. Using a two-wave, multilevel data set (*N* = 16,893, 8481 male; 8412 female; mean age at Time 1: 14.0 years) from 1308 classes in 252 secondary schools in Germany, main effects of the classroom level of prosocial behavior, cross-level interactions between the classroom and the individual levels of prosocial behavior at Time 1, and the moderating role of gender were examined. The results showed that adolescents in classrooms with high collective levels of prosocial behavior at Time 1 reported more prosocial behavior at Time 2, about two years later, reflecting a class-level main effect. A significant cross-level interaction indicated that a high classroom level of prosocial behavior particularly affected individuals with lower levels of prosocial behavior at Time 1. The influence of same-gender peers was larger compared with opposite-gender peers. The findings are discussed with respect to social learning mechanisms in the development of prosocial behavior and their implications for interventions to promote prosocial behavior.

## Introduction

Children and adolescents spend many hours a day interacting with same-aged peers in the school context, and considerable research effort has been directed at investigating the short- and long-term effects of these interactions. For example, a broad literature has examined the spreading of aggressive behavior in peer groups (e.g., Busching and Krahé 2015; Laninga-Wijnen et al. 2019). However, reviews have concluded that there is a lack of longitudinal studies focusing on the positive effects of peer interactions, for example in promoting prosocial behavior. Prosocial behavior is defined as behavior intended to benefit another, which includes helping, donating, sharing, and comforting (Eisenberg et al. 2015). Such studies are needed because although prosocial behavior and aggressive behavior are inversely related, they are not merely two opposite anchors of the same construct, but represent independent dimensions underlying adolescent friendship relations (Farrell et al. 2017). Therefore, the present study investigated how the development of adolescents’ prosocial behavior is shaped by the prosocial behavior of the peers in their classroom, applying multilevel analysis to data from a two-wave longitudinal study.

### The Influence of Peers on the Development of Prosocial Behavior

During adolescence, the primary caregivers become less important, and peers gain more and more influence as socializing agents (Lam et al. 2014). A broad research literature has demonstrated the influence of peers on adolescents’ behavior in a variety domains. The focus of this research has been on problem behaviors, such as smoking and drinking (Ragan 2020; Vitória et al. 2020), problematic social media use (Marino et al. 2020), and aggressive and antisocial behavior (Jung et al. 2019).

Evidence on how the prosocial behavior of peers affects the development of individual prosocial behavior is scarce by comparison. A study found that young adolescents (aged 12-14 years) changed the probability that they would show a broad range of prosocial behaviors in the direction of the (fictitious) probabilities of showing these behaviors indicated by others, including both peers and adults (Foulkes, Leung, Fuhrmann, Knoll, and Blakemore, 2018). Another study found a significant association between the prosocial behavior of adolescents and their friends (Farrell et al. 2017). In a longitudinal study, the degree to which their best friends engaged in prosocial behavior predicted adolescents’ pursuit of prosocial goals, which in turn predicted their prosocial behavior, especially when the affective quality of the friendship and the interaction frequency were high (Barry and Wentzel 2006). In a cross-sectional study with Dutch adolescents, positive correlations between best friends’ engagement in voluntary work and participants’ readiness to volunteer were found (van Goethem et al. 2014). Also studying volunteering as a form of prosocial behavior, an experimental study showed that the impact of prosocial peer behavior on adolescents’ prosocial behavior was moderated by the perceived social status of the peer (Choukas-Bradley et al. 2015). This study used a chat-room paradigm in which participants communicated about volunteering in response to different scenarios with three peers from their grade, who were introduced as being high vs. low in social status. After exposure to the volunteering behavior of the peers, participants showed more prosocial behavior compared to a baseline assessment, but only if they believed the peers had a high social status. The increase remained significant in a subsequent phase when participants indicated their willingness to volunteer in private, knowing that it would not be communicated to others.

### Studying Peer Effects on Prosocial Behavior in Classroom Communities

There is plenty of evidence that children and adolescents choose friends who are similar to themselves, including homophily with regard to prosocial behavior (Shin et al. 2019). Therefore, observed similarity among friends may be due to selection effects, socialization effects, or a combination of both. A three-wave longitudinal study of peer influences on adolescents’ smoking behavior used a cross-lagged design to separate selection from socialization effects and found evidence for both paths (Vitória et al. 2020). Adolescents’ smoking behavior in their younger cohort, similar in age to the present sample, predicted the choice of friend who smoked at Time 2, and friends’ smoking behavior at Time 2 predicted participants’ smoking behavior at Time 3. Neither selection nor socialization effects were found for same-grade students who were not friends. However, classroom effects were treated as control variables in this analysis and not examined in their own right as main effects or in interactions. A two-wave study from South Korea did not find an influence of classmates on prosocial behavior as assessed by teacher reports (Shin 2017).

In a sample of 51 classes, the overall level of prosocial behavior did not predict later prosocial and aggressive behavior of the individual class members (Laninga-Wijnen et al. 2018). However, when classroom characteristics were taken into account, a significant increase in prosocial behavior was found in classes in which there was an association between prosocial behavior and social status. This finding provides indirect evidence for the importance of normative beliefs at the classroom level because it indicates that only in classrooms where prosocial behavior was linked to high status did classroom prosociality affect the individual development of prosocial behavior. Further studies have shown individual prosocial behavior to be more closely linked with social status in classes with a high level of overall prosocial behavior compared to classes with a lower level of prosocial behavior (Dijkstra and Gest 2015; Torrente et al. 2014). However, other studies did not support this relationship (Boor-Klip et al. 2017; Stormshak et al. 1999).

The question whether the effect of the overall level of prosocial behavior in a classroom is moderated by individual differences in prosocial behavior has received little attention so far. Analyses of aggressive behavior have shown that initially nonaggressive students were influenced to a greater extent than initially more aggressive individuals by the collective level of aggression in their class (Busching and Krahé 2015; Rohlf et al. 2016). As parallel processes of observational learning may be assumed for negative and positive forms of social behavior, this evidence may be used to predict that initially less prosocial individuals would be influenced by the prosocial behavior of their peers to a greater extent than individuals with a higher initial level of prosocial behavior.

To provide a test of peer socialization effects on prosocial behavior, the present study was conducted in a setting in which selection effects are minimized and peer groups stay together over an extended period of time. The peers with whom adolescents arguably interact most in terms of time spent together are their classmates (Dishion 2014). Moreover, in contrast to their choice of friends, adolescents have no influence over the peers with whom they are in the same class, as students are assigned to classrooms by the school administration. This provides an excellent research opportunity for investigating the socializing effect of stable peer groups in which selection effects are minimized.

To investigate how the collective behavior of the members of a classroom shapes the development of individual students, the most suitable approach is multilevel analysis. In this approach, it is possible to disentangle effects due to individual characteristics, effects due to the classroom as a whole, and their interactions (Hox et al. 2017). Several studies have used multilevel analysis to demonstrate that peers who are surrounded by aggressive classmates also show more aggressive behavior over time (Henry et al. 2000; Henry et al. 2004). However, classmates’ behavior can also be considered as a resource. Some studies have shown that in classrooms with a higher level of prosocial behavior, individual students show less aggressive and antisocial behavior over time (Hofmann and Müller 2018) and show fewer teacher-rated problem behaviors (O’Brennan et al. 2014), but another study found no effect of the classroom level of prosocial behavior, assessed through peer nominations, on either class members’ aggressive behavior or their victimization by other classmates (Mercer et al. 2009).

The organization of secondary education in Germany provides the structural requirements for investigating peer influences in classroom communities from a longitudinal perspective. Schools typically have a number of parallel classes in each year, and students are assigned to a class by the school authorities without a say by the students themselves and their parents. Classes stay together for several consecutive school years, and students spend most of the school day in the community of their class. In combination, these features mean that self-selection into classroom peer groups is minimized, and classrooms provide a stable context for social learning experiences to occur. Although students are, of course, free to selectively interact with the other students in their class based on friendship and shared interests, they are nonetheless exposed to the behavior of all students in their class over extended periods of time. Therefore, studying the impact of the collective level of prosocial behavior within classrooms is a meaningful approach for understanding peer influences on the development of prosocial behavior. The measure of prosocial behavior used in this study was specifically designed to capture behavior shown in a school context so that it would be observable by all members of the class.

### Social Learning as a Basis for Peer Influences on (Pro)Social Behavior

The psychological processes underlying the influence of peer groups on social behavior in general and prosocial as well as aggressive behavior in particular may be explained by social learning theory (Bandura 1977). This theory highlights observational learning as a key mechanism by which individuals acquire both positive and negative forms of social behavior (van Hoorn et al. 2016). Observational learning is not limited to the acquisition of patterns of behavior but also contributes to the development of social cognitions, such as cognitive scripts and normative beliefs about certain types of behavior (Huesmann 2018). For example, a longitudinal study showed that adolescents became more aggressive over time when they were surrounded by classmates who believed that aggression was an acceptable form of resolving interpersonal conflicts (Busching and Krahé 2015). In classes with a high proportion of aggressive students, aggressive behavior was found to be positively linked to popularity, which contributed to the normative acceptance of aggression in the class over time (Laninga-Wijnen et al. 2020). The link was reduced the higher the number of prosocial students in the class. In a study on intergroup contact between Catholics and Protestants in Northern Ireland, adolescents who perceived their peers to hold positive views about intergroup contact, defining a pro-outgroup norm, were more likely to engage in prosocial behavior toward outgroup members (McKeown and Taylor 2018).

In the case of prosocial behavior, the norm of reciprocity plays a crucial role. The norm of reciprocity refers to the belief that individuals should help others if these had helped them (Penner et al. 2005). Thus, being surrounded by prosocial peers, from whom individuals receive help, should increase their willingness to show prosocial behavior towards these others. This norm should be more salient if many adolescents show prosocial behavior and should facilitate the adoption of prosocial behavior through the process of observational learning. Social learning theory posits that similar models are more likely to serve as a source of observational learning than dissimilar models, so age-homogeneous groups, such as classroom communities, should facilitate observational learning. A three-wave study with young adolescents supports this line of reasoning by showing that Time 1 reports of receiving help from classmates predicted prosocial behavior to classmates at Time 2, which in turn predicted help received from classmates at Time 3 (Stotsky et al. 2019).

### Gender Differences in Prosocial Behavior and Peer Influences

Several studies have shown gender differences in the level and development of prosocial behavior in adolescence. A peer nomination study found that girls were more often named as helpers than boys (van Rijsewijk et al. 2016), and there is evidence that the peak in prosocial behavior is higher and reached earlier in girls than in boys (van der Graaff et al. 2018).

Few studies have investigated gender as a moderator of peer influence effects. Early experimental studies of prosocial behavior found that the impact of a role model showing prosocial behavior was greater on girls than on boys (Grusec and Skubiski 1970). More recent studies have shown gender differences in the appreciation of prosocial behavior. While girls prefer friends with similar levels of prosocial behavior to their own, prosocial behavior does not seem to play a role in friendship selection for boys (Hsiao et al. 2019). However, in the study by Boor-Klip et al. (2017), the relationship between individual prosocial behavior and social status was closer for boys than for girls.

Three different possibilities for gender to affect social influence in peer groups may be distinguished (Brechwald and Prinstein 2011): (1) the gender of the target person, for example whether girls are more easily influenced than boys. Relevant to this question is a finding that boys were more susceptible than girls to peer pressure to engage in antisocial behavior, but no such difference was found for prosocial behavior (Farrell et al. 2017); (2) the gender of the influencer, for example whether girls as a group have a stronger impact on their classmates than boys as a group. Such a main effect of influencer gender was observed for aggressive behavior in a study that found girls to have a greater impact as a group compared to boys on both their male and their female classmates (Busching and Krahé 2015); (3) the interaction of influencer and target gender, for example whether individuals are more influenced by same-gender than by opposite-gender peers. The latter possibility is suggested by the finding that more than 80% of the nominated helpers had the same gender as the nominating person (van Rijsewijk et al. 2016). To address these potential moderation effects, separate class-level scores of prosocial behavior at T1 based on the male and female class members, respectively, were calculated in the present study.

## The Current Study

To investigate the impact of classroom prosocial behavior on the development of prosocial behavior in individual students over time, the current study was designed to test two predictions. The first prediction was that individuals would show more prosocial behavior over time if they were in a class in which the overall level of prosocial behavior was high than if they were in a class with a lower overall level of prosocial behavior (Hypothesis 1). This hypothesis predicts a main effect of the classroom level of prosocial behavior on individual class members.

Based on previous evidence concerning aggressive and deviant behavior, the second prediction was that classroom effects of prosocial behavior would be more pronounced on class members with lower prosocial behavior at T1 (Hypothesis 2). Specifically, it was expected that the initially less prosocial adolescents in a prosocial classroom would show a greater increase in prosocial behavior over time than those in the same classroom who were more prosocial to begin with. This hypothesis postulates a cross-level interaction between classroom level and individual level of prosocial behavior at T1 on individual prosocial behavior at T2. Multilevel analysis was employed as the correct approach for testing these hypotheses. This approach partitions the dependent variable into variation due to individual class members (individual level), differences between the classrooms (class level), and differences between schools (school level).

In addition to these two hypotheses, the present study investigated the role of gender as a potential moderator of classroom effects on prosocial behavior. Because results from past studies have been inconclusive, this issue was addressed as a research question rather than a directed hypothesis. Because gender was treated as a binary construct in the current data set, gender effects could only be examined for males and females.

## Method

### Participants and Procedure

Participants represented a subgroup of a large cross-sectional and longitudinal data set with up to three measurement points collected in Germany (StEG-Konsortium 2011)^1^. Information about which class they attended was available for all participants. All classes that participated in at least two data waves were included in the sample. If a class participated three times, two neighboring time points were chosen at random. This led to a sample of *N* = 24,334 at T1, and *N* = 21,476 at T2 in 1,308 classes in 252 schools. Participation rates (i.e., the percentage of students on the school student register who participated in the study) ranged from 76.8% to 79.7% per data wave. A total of 18,659 students took part in both measurement points. The main reasons for non-participation were absence on the day of testing and lack of parental consent. Of these,1766 (9%) were excluded because they had missing data. The final sample consisted of 16,893 students (8481 male and 8412 female), who participated in both measurement points without missing data. The mean age of the sample was 14.0 years (*SD* = 1.20; range: 9–16 years) at T1, and girls were on average one month older than boys. The mean number of students per class was 12.91 (*SD* = 5.70). The mean interval between the two data waves was 22.4 months (*SD* = 4.8 months).

Ethics approval was obtained from an independent scientific advisory board overseeing the original data collection and from the school authorities of the participating federal states. Data collection took place in class during school hours between 2005 and 2009. Each testing session lasted for about 90 minutes, including a short break. Parental consent was obtained for each participant.

### Instruments

#### Prosocial behavior

Prosocial behavior was assessed by five items: (1) “I helped classmates with learning or homework”; (2) “I helped new classmates to settle into the school”, (3) “I helped to make sure our desks and classrooms remained clean”; (4) “I helped classmates to resolve a quarrel without violence”; (5) “I took action to calm the class down when someone showed disruptive behavior”. Participants were asked how often they had shown these behaviors on a scale from 1 (*never*) to 5 (*almost every day*) in the last twelve months. The internal consistency of the scale was good (alpha_T1_ = 0.72, alpha_T2_ = 0.73). The measure was used in a previous study based on a different subset of participants from the same data set and found to be reliable (Sauerwein et al. 2016). Evidence of the validity of the measure is presented in the Results section.

#### Perspective taking and empathy

The four-item scale “Perspective taking and empathy” was used to provide evidence for the construct validity of the prosocial behavior measure. The four items were (1) “In a disagreement, I try to look at the issue from the perspective of all parties before I takes sides”; (2) “I believe that every problem has two sides, and I try to look at both”; (3) “I sometimes try to understand my friends better by imagining how things look from their point of view”; (4) “Before I criticize people, I try to imagine how I would feel if I were in their position”. The internal consistencies of the scale were alpha_T1_ = 0.66, and alpha_T2_ = 0.75). The mean scores were *M* = 2.90 (*SD* = 0.67) at T1 and *M* = 2.84 (*SD* = 0.66) at T2.

#### Control variables

Four control variables were included in all hypothesis-testing analyses to account for the potential impact of third variables on the paths in the multilevel models: age and academic achievement as continuous control variables, school track and migration background as categorical control variables. Age was included to account for the variation in age in the sample, which ranged from 9 to 16 years. School track was included because the German secondary school system is divided between schools with an academic orientation, leading to university entrance qualifications, and vocational schools, leading to, or preparing for, qualifications for entering vocational training. Students from lower socioeconomic backgrounds tend to be underrepresented in the academically oriented type of school. Therefore, it was necessary to control for differences in school track in the analyses. The exact number and names of the lower tracks differ between the federal states in Germany. For the purposes of the present analysis, a dichotomous score was created in which the highest level was called the academic track, and all tracks below were combined into a vocational track, in line with previous research (Krahé et al. 2013). Migration background is linked to school track, with students from a migration background being underrepresented in academically oriented schools. Participants were asked whether they, their father, and/or their mother had not been born in Germany. If they answered yes to at least one of these three questions, they were coded as having a migration background. The fourth control variable was academic achievement, which is a relevant variable related to prosocial behavior in a school context (e.g., Gerbino et al. 2018). The mean of the grades in the core subjects of Math, German, Geography, and the first foreign language on the latest report card, as reported by the student, was calculated to yield a score of academic achievement. In Germany, the grading scale ranges from 1 (*very good*) to 6 (*insufficient*). For ease of interpretation, the grades in the four subjects were averaged, and the mean score was then reverse-coded so that higher scores indicate higher academic achievement. Finally, gender was included as an additional control variable in the analyses for the sample as a whole.

### Data Preparation and Analysis

Five different scores were calculated for all participants: (1) the mean of the prosocial behavior of all students in their class, representing the overall classroom level of prosocial behavior. For these scores, all students who were present at the respective data wave (*N* = 24,334 at T1, *N* = 21,476 at T2) were included because they were part of the regular class environment; (2) the individual’s deviation from the class mean, representing the individual score relative to the classroom mean, for the 16,893 students in the final sample; (3) the mean of the girls’ prosocial behavior in their classroom, (4) the mean of the boys’ prosocial behavior in their classroom, and (5) the individual’s deviation from the mean of their same-gender classmates. Moreover, we included the school as a third level to control for any selection effects at this level due to third variables (e.g., parental education, neighborhood characteristics), because parents have a certain degree of choice to which secondary school they send their child. The analyses were performed in R with the lme4 package. Significance testing was conducted using Bayesian significance testing of conventional computation of standard errors. Since there are no measures of effect size for multilevel models including cross-level interactions and random components, we additionally report beta weights, which provide an indication of the size of the effects (Peterson and Brown 2005).

To test the sensitivity of our analytic approach, we further inspected the results using parametric bootstrapping with 10,000 replications. Additionally, we estimated all models without control variables. The results of these additional analyses fully confirmed the results of the main analyses and can be found in the Supplementary Material (Tables S1–S3). Post-hoc-tests were conducted using the simple-slope technique.

## Results

### Validation of the Prosocial Behavior Measure

Because no information about the validation of the prosocial behavior measure was available, we tested the factorial structure as well as the correlation of prosocial behavior with related constructs (Hussey and Hughes 2020). Confirmatory factor analyses showed a good fit of a single-factor model at T1, *χ*^2^(5) = 324.78, TLI = 0.953, CFI = 0.977; RMSEA = 0.052 95% CI = [0.47; 0.057], and T2, *χ*^2^(5) = 239.38, TLI = 0.982, CFI = 0.965; RMSEA = 0.047 95% CI = [0.042; 0.052]. To assess measurement invariance over time, we first tested a model specifying weak measurement invariance. After allowing two items to correlate between T1 and T2, the model fit was good, indicating that the pattern of item loadings did not change over time, *χ*^2^(37) = 1363.03, TLI = 0.952, CFI = 0.960; RMSEA = 0.046 95% CI = [0.47; 0.057]. Strong measurement invariance additionally requires that the intercepts of the indicator items are equal over time. The fit of this more constrained model was acceptable *χ*^2^(41) = 1603.71, TLI = 0.949, CFI = 0.949; RMSEA = 0.037 95% CI = [0.036; 0.039]. The correlation between the latent factors of prosocial behavior at T1 and T2 was *r* = 0.49 (*z* = 45.32, *p* < 0.001), which is highly similar to the coefficient reported in earlier research for a two-year interval (*r* = 0.48) (Coyne et al. 2018). Because most studies testing construct stability use a one-year interval instead of the two-year interval covered in the present study, it seems appropriate to translate the coefficient of stability into the value that would be expected for a one-year interval, which yielded a value of *r* = 0.70 (*z* = 90.76, *p* < 0.001).^2^

Finally, a significant positive correlation with perspective taking at T1 (*r* = 0.32, *t*(22252) = 49.81, *p* < 0.001) and T2 (*r* = 0.33, *t*(20894) = 50.861, *p* < 0.001) can be seen as evidence of construct validity. The finding that girls showed more prosocial behavior compared to boys may also be interpreted as evidence of the validity of the measure, because the size of the gender difference (T1: *d* = 0.20; T2: *d* = 0.25) is in the same range as the effect size of a meta-analysis (Fabes et al. 1999).

### Descriptive Statistics and Correlations

The means, standard deviations, and correlations for all variables at the classroom level and the individual level are presented in Table 1. It is worth noting that the mean score of prosocial behavior at the class level was below the midpoint of the response scale. This means that there is no ceiling effect that would limit the potential for an increase in prosocial behavior over time among those participants starting at a relatively higher level of prosocial behavior at T1. The overall classroom level of prosocial behavior was highly correlated with the gendered classroom-level scores among girls and boys at T1 and T2. However, the correlation between the girls’ and the boys’ level of prosocial behavior was only in the medium range, indicating that the two gender groups may provide different learning opportunities within their class. The correlation between gender and prosocial behavior indicates that girls reported more prosocial behavior than did boys. In addition, a small positive correlation of individual prosocial behavior with age was found.

**Table 1:** Means, standard deviations, and correlations

Construct	*M* (*SD*)	1	2	3	4	5	6	7	8	9	10	11	12	13
**Class-level constructs**														
1. All class members’ level of prosocial behavior (T1)	2.42 (0.36)	–												
2. Girls’ classroom level of prosocial behavior (T1)	2.51 (0.45)	0.81												
3. Boys’ classroom level of prosocial behavior (T1)	2.32 (0.43)	0.83	0.40											
4. All class members’ level of prosocial behavior (T2)	2.21 (0.32)	0.54	0.45	0.43										
5. Girls’ classroom level of prosocial behavior (T2)	2.32 (0.40)	0.42	0.43	0.26	0.79									
6. Boys’ classroom level of prosocial behavior (T2)	2.11 (0.38)	0.45	0.29	0.45	0.80	0.34								
**Individual-level constructs**														
7. Academic track^1^	1.78 (0.41)	0.05	0.04	0.07	−0.10	−0.05	−0.08							
8. Individual prosocial behavior (T1)	0.00 (0.86)	−0.00	0.00	−0.01	0.01	0.01	0.01	0.00						
9. Individual prosocial behavior (T2)	0.00 (0.75)	0.01	0.01	0.00	0.00	0.01	−0.00	−0.00	0.33					
10. Gender^2^	1.52 (0.50)	−0.01	−0.00	0.03	−0.01	0.01	0.02	0.04	−0.11	−0.13				
11. Migration background	1.71 (0.45)	−0.18	−0.14	−0.15	−0.18	−0.16	−0.13	−0.06	−0.05	−0.05	0.01			
12. Age	0.00 (1.00)	−0.33	−0.28	−0.25	−0.20	−0.14	−0.18	0.15	0.02	0.01	0.04	−0.04		
13. Acad. achievement	−2.91 (0.76)	0.02	0.02	−0.01	0.03	0.01	0.02	−0.26	0.04	0.06	−0.08	0.14	−0.26	

School track: 1 = vocational, 2 = academic; gender: 1 = female, 2 = male. Because of the multilevel structure, conventional significance testing is misleading. Therefore, no *p* values are indicated

Table 2 presents the intraclass correlation coefficients (ICC). The ICCs indicate how much variance of a variable is explained at the individual level, the classroom level, and the school level. While most of the variance was at the individual level, there were small but significant amounts of variance in prosocial behavior explained at the classroom level. Compared to ICCs commonly encountered in school settings (Hedges and Hedberg 2007), the variance at the classroom level is lower in the present data. This may be due to the fact that prosocial behavior is not an explicit part of the school curriculum, unlike most of the variables commonly investigated in school settings. Additional variance is explained by the school, as school populations vary as a function of sociodemographic and community variables. Therefore, we included the school level as a control variable in the analysis, while focusing on the classroom level as the theoretical construct of interest.

**Table 2:** Intraclass correlation coefficients (ICCs) of prosocial behavior

Time	Group	Individual level	Class level	School level
T1	All	0.90 [0.89; 0.92]	0.06 [0.05; 0.07]	0.03 [0.02; 0.04]
T1	Girls	0.89 [0.88; 0.91]	0.08 [0.06; 0.09]	0.03 [0.02; 0.04]
T1	Boys	0.90 [0.88; 0.91]	0.07 [0.05; 0.08]	0.04 [0.03; 0.05]
T2	All	0.91 [0.89; 0.92]	0.04 [0.03; 0.04]	0.06 [0.05; 0.07]
T2	Girls	0.88 [0.87; 0.90]	0.05 [0.04; 0.07]	0.06 [0.05; 0.08]
T2	Boys	0.91 [0.90; 0.93]	0.05 [0.03; 0.06]	0.04 [0.03; 0.06]

All coefficients are significantly different from zero. 95% CI in brackets

### Main Effects of Classroom Prosocial Behavior and Cross-Level Interactions

To test Hypothesis 1, predicting a main effect of the classroom level of prosocial behavior on individual class members’ prosocial behavior at T2, a multilevel model was estimated. The dependent variable was individual prosocial behavior at T2, and the predictors were the individual as well as the classroom-level prosocial behavior scores at T1. Academic track, academic achievement at T1, migration background, age, and gender were included as control variables. To account for unexplained classroom effects and school differences, random components were included for the classroom as well as the school levels.

The coefficients can be found in Table 3 in the column Model 1. Consistent with Hypothesis 1, the classroom level of prosocial behavior at T1 significantly predicted individual prosocial behavior at T2, b_class level_ = 0.44, 95% CI = [0.39; 0.48], *p* < 0.001. This means that individuals who were in a class with a high level of prosocial behavior at T1 reported more prosocial behavior at T2 compared to individuals who were in classes with lower levels of prosocial behavior at T1. In addition, individual prosocial behavior at T2 was significantly predicted by the corresponding score at T1, b_individual_ = 0.29, 95%, CI = [0.27; 0.30], *p* < 0.001. The classroom-level coefficient is significantly larger than the individual-level coefficient, which shows that the group level is a better predictor of later individual behavior than the individual’s starting level of prosocial behavior. Additionally, higher age, b_age_ = 0.02, 95% CI = [0.01; 0.03], *p* = 0.01, and higher academic achievement at T1, b_academic achievement_ = 0.06, 95% CI = [0.04; 0.08], *p* = 0.004, predicted a higher level of prosocial behavior about two years later. Of the control variables, gender and academic track significantly predicted prosocial behavior at T2.

**Table 3:** Multilevel models predicting T2 prosocial behavior

	Model 1				Model 2			
	b	95% CI	ß	*p*	b	95% CI	ß	*p*
Intercept	1.37	(1.25, 1.49)		<0.001	1.36	(1.24, 1.49)		<0.001
Prosocial behavior (class level)	0.44	(0.39, 0.48)	0.19	<0.001	0.44	(0.39, 0.48)	0.19	<0.001
Prosocial behavior (individual level)	0.29	(0.27, 0.30)	0.30	<0.001	0.53	(0.43, 0.64)	0.56	<0.001
Gender	−0.07	(−0.08, −0.06)	−0.09	<0.001	−0.07	(−0.08, −0.06)	−0.09	<0.001
Age	0.02	(0.01, 0.03)	0.02	0.01	0.02	(0.01, 0.03)	0.02	0.01
Migration background	−0.05	(−0.06, −0.03)	−0.05	<0.001	−0.05	(−0.06, −0.03)	−0.05	<0.001
Academic achievement	0.06	(0.04, 0.08)	0.05	<0.001	0.06	(0.04, 0.08)	0.05	<0.001
Academic track	−0.04	(−0.06, −0.01)	−0.04	<0.001	−0.04	(−0.06, −0.01)	−0.04	<0.001
Prosocial behavior (class level* individual level)					−0.10	(−0.14, −0.06)	−0.26	<0.001

*p < 0.05

**Fig. 1:**
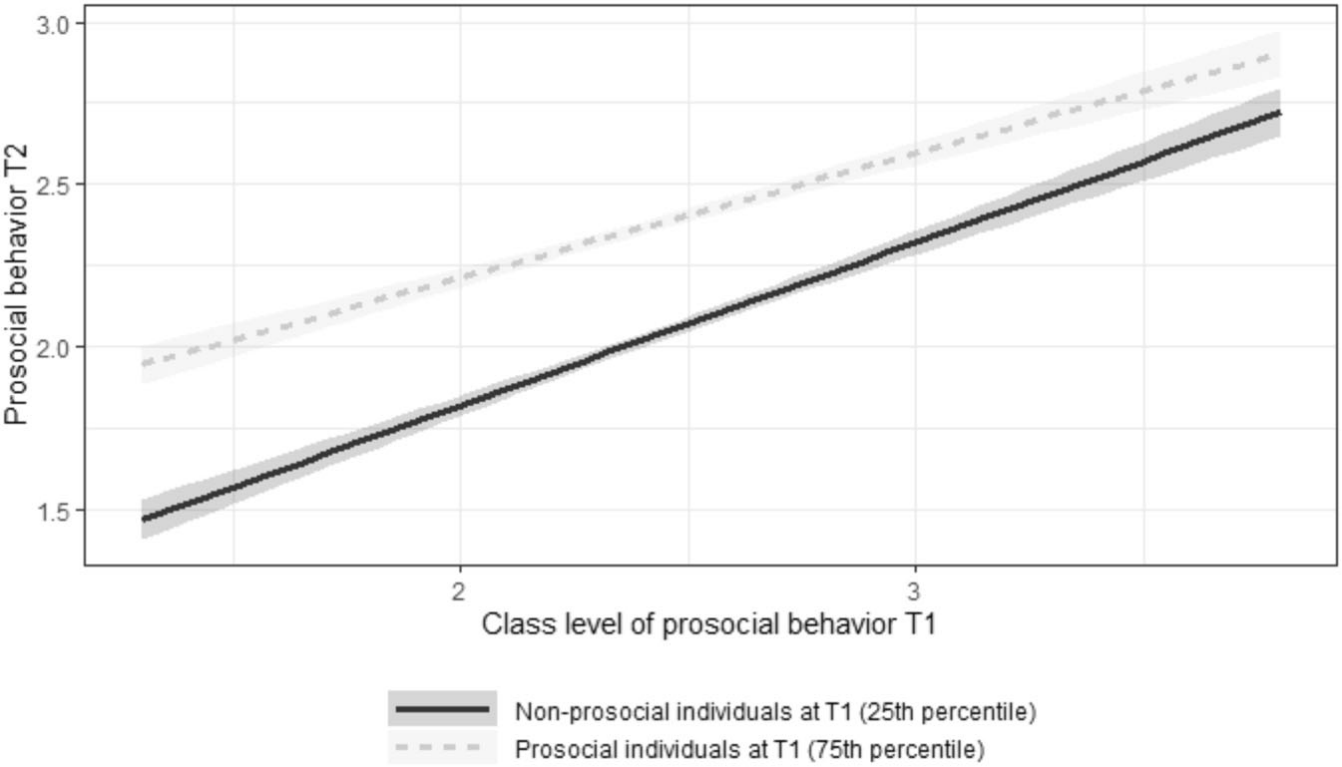
Plot of the interaction between class-level and individual-level prosocial behavior

Hypothesis 2 predicted that the effect of classroom-level prosocial behavior would be stronger for individuals with low as compared to high prosocial behavior at T1. To test this hypothesis, the multilevel model was extended by including the interaction between classroom-level and individual-level prosocial behavior at T1 (Table 3, column Model 2). A significant cross-level interaction was found, b = −0.10, 95% CI = [−0.14, −0.06], *p* < 0.001, which is shown in Fig. 1. The impact of the classroom level of prosocial behavior was stronger on students with a lower level of prosocial behavior at T1 than on those with a higher level of prosocial at T1, consistent with Hypothesis 2.

### Gender Effects

An open research question was formulated regarding gender effects. Three possible ways in which gender might operate as a moderator of classroom effects on individual development were tested: (1) one of the gender groups may be more susceptible to classroom effects than others, (2) one group may be more influential than the other, or (3) individuals may be more influenced by same-gender than by opposite-gender classmates. To address these possibilities, we calculated separate classroom-level means for boys and girls in each class. As a gendered individual-level predictor, we calculated the deviation of each participant’s score from their same-gender classmates. Additionally, we included the cross-level interactions between the individual-level score and participant gender and the two gendered classroom-level means. The control variables as well as the random effects were the same as in the models before. The results are shown in Table 4.

**Table 4:** Multilevel model predicting T2 prosocial behavior by gendered prosocial scores at T1

Predictor	b	95% CI	ß	*p*
Intercept	1.39	(1.28, 1.51)		<0.001
Girls’ classroom level of prosocial behavior (T1)	0.22	(0.19, 0.26)	0.12	<0.001
Boys’ classroom level of prosocial behavior (T1)	0.20	(0.17, 0.24)	0.11	<0.001
Individual prosocial behavior (T1)	0.54	(0.43, 0.64)	0.54	<0.001
Gender	−0.08	(−0.16, −0.01)	−0.10	0.04
Age	0.02	(0.004, 0.03)	0.02	0.02
Migration background	−0.05	(−0.06, −0.04)	0.05	<0.001
Academic achievement	0.06	(0.04, 0.08)	0.05	<0.001
Academic track	−0.03	(−0.06, −0.01)	−0.04	0.01
Girls’ classroom level of prosocial behavior (T1)	−0.04	(−0.08, −0.004)	−0.12	0.03
* Individual prosocial behavior (T1)				
Boys’ classroom level of prosocial behavior (T1) * Individual	−0.06	(−0.10, −0.02)	−0.14	<0.001
prosocial behavior (T2)				
Girls’ classroom level of prosocial behavior (T1) * Gender	−0.15	(−0.18, −0.12)	−0.47	<0.001
Boys’ classroom level of prosocial behavior (T1) * Gender	0.15	(0.12, 0.18)	0.45	<0.001
Individual prosocial behavior (T1): Gender	−0.02	(−0.01, 0.01)	−0.02	0.68
Girls’ classroom level of prosocial behavior (T1) * Individual	0.01	(−0.02, 0.05)	0.04	0.41
prosocial behavior (T1) * Gender				
Boys’ classroom level of prosocial behavior (T1) * Individual	−0.01	(−0.04, 0.03)	−0.02	0.72
prosocial behavior (T1) * Gender				
Observations	16,707			
Log Likelihood	−18,518.04			
Akaike Inf. Crit.	37,076.08			
Bayesian Inf. Crit.	37,230.55			

The *N* is slightly lower than the total *N* of 16,893 because for a small number of classes that contained only male or female students, no gendered scores could be computed

*p < 0.05

**Fig. 2:**
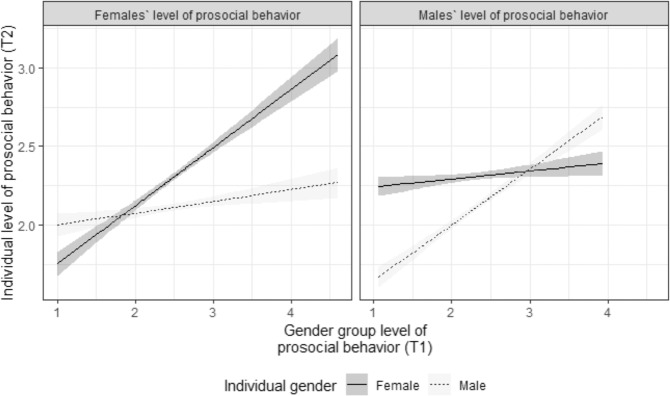
Interaction between girls’ and boys’ classroom levels of prosocial behavior and participant gender

Significant cross-level interactions were found between the two gendered class-level scores of prosocial behavior and participant gender, as shown in Fig. 2. To further explore these interactions for each gender group, we calculated the respective regression weights using the simple-slope technique developed by Aiken and West (1991). These post-hoc analyses showed that girls were more influenced by the prosocial behavior of the other girls in their class, b_Girls*female prosocial behavior_ = 0.37, 95% CI = [0.32; 0.42], *p* < 0.001, whereas boys were more influenced by the prosocial behavior of the other boys, b_Boys*male prosocial behavior_ = 0.36, 95% CI = [0.31; 0.41], *p* < 0.001. In addition, opposite-gender classmates also had an effect. Girls reported a higher level of prosocial behavior two years later if they had been surrounded by more (rather than less) prosocial boys, b_Girls*male prosocial behavior_ = 0.08, 95% CI = [0.03; 0.12], *p* < 0.001, and boys reported a higher level of prosocial behavior if they had been surrounded by more (rather than less) prosocial girls, b_Boys*female prosocial behavior_ = 0.05, 95% CI = [0.005; 0.10], *p* = 0.04. Using the calculated confidence intervals, it is possible to compare the two coefficients. They can be regarded as statistically significant if their confidence intervals do not overlap. This comparison indicates that the cross-gender coefficients were significantly smaller than the same-gender coefficients for both gender groups, girls_same-sex_: b = 0.37; 95% CI [0.32–0.42] vs. girls_opposite-sex_ b: = 0.08, 95% CI [0.03–0.12]; boys_same-sex_: b = 0.36; 95% CI [0.31–0.41] vs. boys_opposite-sex_: b = 0.05, 95% CI [0.005–0.10]. Neither the two same-gender coefficients nor the two cross-gender coefficients differed significantly from each other. To summarize, the findings show that both same-gender and opposite-gender peers affected the development of prosocial behavior of girls and boys, but the impact of same-gender peers was stronger.

**Fig. 3:**
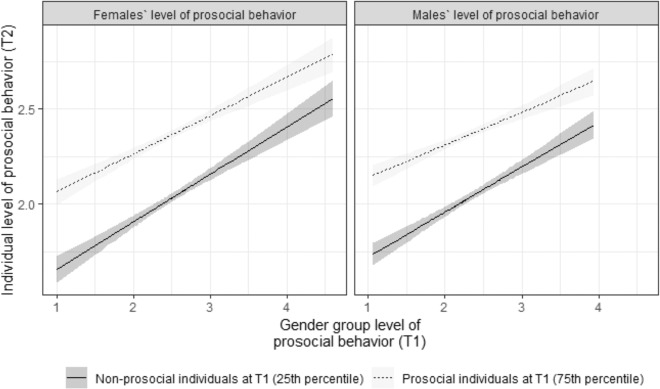
Interaction between individual prosocial behavior and the girls’ (left panel) as well as the boys’ (right panel) classroom levels of prosocial behavior

The last two significant cross-level interactions reflect the moderating effect of individual prosocial behavior at T1 on the impact of gendered classroom scores of prosocial behavior, b_Girls’ level of prosocial behavior*individual prosocial behavior_ = −0.04, 95% CI = [−0.08, −0.004]; b_Boys’ level of prosocial behavior*individual prosocial behavior_ = −0.06; 95%, CI = [−0.10, −0.02). The interactions are plotted in Fig. 3. In line with the findings for the class as a whole, the gendered analysis showed that being in a class in which the same-gender peers reported a higher level of prosocial behavior affected initially less prosocial individuals more than it affected individuals with higher levels of prosocial behavior at T1. This finding held for both girls and boys, as the three-way interaction between gendered class-level scores, individual prosocial behavior at T1, and participant gender was nonsignificant.

## Discussion

The development of prosocial behavior is a critical challenge in adolescence. Social learning theory conceptualizes prosocial behavior as a form of social behavior that is learned in the course of socialization, not only through direct reinforcement, but also through observational learning (Bandura 1977). Adolescents spend an extensive amount of time in the company of their peers, with ample opportunities for observational learning. Although past research has mainly focused on peer influences on the development of aggressive and antisocial behavior, positive peer behavior may be conceptualized as a learning resource that operates in a parallel way to affect individuals’ behavior. Accordingly, the current study examined the impact of classroom communities on facilitating the social learning of prosocial behavior in adolescence.

Exploiting the fact that the German school system minimizes self-selection into classrooms, and classroom communities remain together over consecutive school years, data from a large nationwide sample were used to test two hypotheses: The first hypothesis predicted that individuals surrounded by classmates with a higher collective level of prosocial behavior would be more prosocial about two years later than individuals surrounded by less prosocial classmates. The second hypothesis predicted that the impact of classmates’ prosocial behavior would be stronger on individuals starting off with a lower level of prosocial behavior than for individuals already reporting high prosocial behavior at the first data wave. Both predictions received support by the findings, consistent with the view that peers are influential socialization agents in adolescence. Whereas the negative influence of peers in increasing problem behavior has been studied extensively, scholars agree that the potential of peers to promote positive social behaviors and outcomes has received insufficient attention in past research (Barry and Wentzel 2006). The present study addressed this gap by demonstrating that individuals who are part of classroom communities with a high collective level of prosocial behavior showed an increase in prosocial behavior over time. In contrast to previous studies that failed to find a main effect of classroom-level prosocial behavior on the development of individual prosocial behavior (e.g., Laninga-Wijnen et al. 2018), the present data showed that individuals’ prosocial behavior increased if they were surrounded by prosocial classmates. A notable difference between the two studies is that the number of classes included in the present study was much higher (1308 vs. 51), yielding a more powerful basis for the estimation of class-level effects.

However, the main effect of the class level was qualified by a significant cross-level interaction with individual levels of prosocial behavior at Time 1. Whereas previous studies found evidence for class characteristics as moderators of class-level effects, such as the degree to which social status or popularity were tied to prosocial or aggressive behavior (Laninga-Wijnen et al. 2020), the present results showed that class-level effects were moderated by individual differences in the form of class members’ initial levels of prosocial behavior. In parallel to findings for aggressive behavior (Busching and Krahé 2015, 2018), being in a prosocial class community had a greater impact on the development of prosocial behavior of the initially less prosocial members than on those members who were more prosocial at the first data wave. Because the mean level of prosocial behavior in the present sample was below the midpoint of the response scale, this finding cannot be attributed to a ceiling effect. It is consistent with social learning theory, which sees the learning of social behavior as a result of both direct and vicarious reinforcement. Because prosocial behavior tends to be rewarded, observing peers’ prosocial behavior being followed by positive consequences should have a greater impact on initially less prosocial individuals because these individuals experience less direct reinforcement in response to their own prosocial actions. A combination of longitudinal and multilevel designs is particularly suited to detect these interactions of collective and individual prosocial behavior and is recommended for use in future studies.

The findings have theoretical as well as practical implications. At a theoretical level, they mirror the pattern of classroom main and cross-level effects found for deviant and aggressive behavior (Busching and Krahé 2015, 2018; Rohlf et al. 2016). This evidence is consistent with a conceptualization of peer influences based on social learning theory, which identifies observational learning and script learning as general mechanisms by which social behavior is shaped through social interactions. These learning principles are thought to operate in the same way across different types of behavior. Therefore, demonstrating similar effects for both anti- and prosocial behavior is a critical result. Future studies should extend these multilevel analyses to other domains of social behavior for which peer influences have been shown to be relevant, such as eating behavior (e.g. Salvy et al. 2012), smoking (e.g. Hoffman et al. 2006), or sexual behavior (Widman et al. 2016).

A noteworthy finding of the present study is that highly prosocial classrooms promoted prosocial behavior in initially less prosocial individuals, but classrooms with a low level of prosocial behavior did not make the initially more prosocial individuals less prosocial over time. This pattern mirrors the findings from previous studies of aggressive behavior, where aggressive individuals placed in classroom communities with low collective aggression did not become less aggressive over time (Busching and Krahé 2015, 2018). The finding that parallel patterns emerged with regard to two different types of social behavior suggests an explanation in terms of a general process rather than a process specific to prosocial behavior. Based on social learning theory, one could argue that classroom effects depend on the behavior being shown as a prerequisite for observational learning to occur. If a behavior is rarely shown, be it prosocial or aggressive, no learning opportunities are provided that could lead to changes in the individual’s behavioral repertoire.

Finally, the findings provide evidence on possible moderating effects of gender. This question has rarely been addressed within a multilevel framework, and past results have been inconsistent. In the present study, the collective prosocial behavior of both same-gender and opposite-gender peers predicted individual prosocial behavior at T2, but the influence of peers of the same gender was stronger. This pattern is consistent with the finding that same-gender friendships are by far more common than cross-gender friendships in adolescence. A study in six European cities found rates of other-gender friendships of only 21% for boys and 13.2% for girls (Grard et al. 2018). It is further in line with evidence that the vast majority of nominations of prosocial peers are made within gender groups (van Rijsewijk et al. 2016). This means that opportunities for learning prosocial behavior from same-gender peers are likely to be greater than learning opportunities involving other-gender peers, even within the context of mixed-gender classrooms.

This moderation effect differs from a previous study with adolescents that investigated aggressive behavior. In that study, both girls and boys were more influenced by the girls’ than by the boys’ normative acceptance of aggressive behavior in their class, and no same-gender effects were found for boys (Busching and Krahé 2015). The authors argued that the finding that girls define the boundaries for the aggressive behavior of girls and boys alike may be due to the increased importance of romantic relationships in adolescence, which may lead boys to conform to the norms about aggression defined by the girls. Why no parallel effect was found for prosocial behavior cannot be explained conclusively on the basis of the current data and needs to be addressed in future studies.

A further task for future studies is to integrate the different forms of pro- and antisocial behavior for which peer group effects have been demonstrated into a common design (Laninga-Wijnen et al. 2019). For example, can a high level of prosocial behavior in a class reduce individual class members’ aggressive behavior over time or is there a risk for a high collective level of aggression in a class to reduce individual class members’ prosocial behavior in a longitudinal perspective? There is some evidence that a high level of prosocial behavior may reduce individuals’ aggression (Hofmann and Müller 2018), but it is yet unclear whether the reverse link can also be found and whether these peer group effects are moderated by individual characteristics.

The findings also have implications for intervention measures designed to promote prosocial behavior. Because a higher level of prosocial behavior is associated with positive outcomes at the group level, such as a better school climate (O’Brennan et al. 2014), seeking to create a high collective level of prosocial behavior in classrooms is an important goal. Based on the present research, increasing the level of prosocial behavior by targeting all students may be expected to raise the overall level of prosocial behavior in the classroom (see Laninga-Wijnen et al. 2020, for a similar argument). As a result, the initially less prosocial students may shift in the direction of the higher level of prosocial behavior in their (Conklin et al. 2017). This could be a better strategy than targeting only the less prosocial students, whose behavior may be harder to change by explicit intervention efforts.

The gender effects found in the present study indicate that both same-gender and other-gender peers are influential, even if the influence of same-gender peers seems to be stronger. Interventions seeking to promote prosocial behavior at the classroom level should seek to address gendered norms and behaviors regarding prosocial acts and address the fact that cross-gender interactions become more important in the course of adolescence.

In evaluating the present findings, both strengths and limitations should be noted. The main strengths are the representativeness of the sample, the large number of classrooms and the use of state-of-the-art multilevel analysis to detect both main effects and cross-level interactions on changes in prosocial behavior from Time 1 to Time 2. A first limitation of this study is that it did not take teachers’ perspectives and behaviors into account. Teachers are an important source for the development of prosocial behavior in classrooms (Jennings and Greenberg 2009), and their reactions toward the overall prosocial behavior in a class could influence students’ behavior. However, if the classroom effects were due to teachers’ behavior, the parallel pattern for aggression and prosocial behavior would be hard to explain, since teachers may be assumed to react differently to the two forms of behavior, encouraging prosocial behavior and discouraging aggression. A second limitation is that prosocial behavior was assessed only by self-reports. However, if social desirability had led to an overreporting of prosocial behavior, this would have only affected the class-level means but cannot explain the class-level effects on individual behavior and the cross-level interactions. Nevertheless, future studies should include alternative measures of prosocial behavior, such as teacher ratings (Krahé and Möller 2011) or peer nominations (Mercer et al. 2009). Furthermore, only prosocial behavior was examined in this study. Social learning and the acquisition of prosocial scripts also involves prosocial norms, which have a strong evaluative component. Previous research on classroom effects on aggressive behavior showed that individual aggressive behavior increased not only in classrooms with a high level of aggressive behavior but also in classes with a high normative acceptance of aggression (Busching and Krahé 2015). From a theoretical point of view, a parallel finding should be expected for shared normative beliefs about prosocial behavior in a classroom. A final limitation is that the data set on which the present findings are based adopted a gender-binary definition that only offered the response categories of male and female. Broadening the analysis of gender effects to include non-binary categories of gender identification is a task for future research.

## Conclusion

The present findings contribute a novel perspective to understanding the role of peer influences on the development of prosocial behavior in adolescence by examining peer group influences with a combined multilevel-longitudinal analysis. The results showed a main effect of the classroom level of prosocial behavior: Consistent with the tenets of social learning theory, adolescents in a classroom with peers who showed a high level of prosocial behavior became more prosocial over time, suggesting that the peer environment offers a resource for the observational learning of prosocial behavior. In addition, the multilevel design enabled us to identify significant cross-level interactions, indicating that the initially less prosocial individuals benefitted more from being surrounded by prosocial classmates than those who were more prosocial to begin with. Because self-selection effects were minimized in the present design, the class-level main effects and interactions may be seen as reflecting a socializing effect rather than a homophily effect, that is the tendency to affiliate with similar peers. The findings suggest that interventions designed to increase the collective level of prosocial behavior as a whole may be a promising strategy for stimulating the social learning of prosocial behavior in classroom communities.

## Supplementary information

Supplementary Materials

## Footnotes

1 The data collection was organized so that a majority of participants were approached only once (*N* = 51,192), because either they had left school or data collection finished after the first wave. The collection of longitudinal data had only been planned for a subsample. A detailed analysis regarding the difference between the cross-sectional and longitudinal samples is presented by Furthmüller (2015).

2 Based on Kline (2011), the following equations were applied: $r_{two\,years} = r_{year_1} \ast r_{year_2}$. Assuming that the stability is constant across the two years ($r_{year_1} = r_{year_2} = r_{one\,year}$), it is possible to transform the equation $r_{two\;years} = r_{one\;year} \ast r_{one\;year} \Leftrightarrow r_{one\;year} = \sqrt {r_{two\;years}}$.
