# Supplementary material for: With a Little Help from Their Peers: The Impact of Classmates on Adolescents’ Development of Prosocial Behavior
Source: J Youth Adolesc. 2020 Jun 11;49(9):1849–63. doi: 10.1007/s10964-020-01260-8 (PMC7423867; doi:10.1007/s10964-020-01260-8)
Supplement: Supplementary file 1 — Supplementary Materials [file 10964_2020_1260_MOESM1_ESM.docx]

**Supplementary Material**

With a Little Help from Their Peers:

The Impact of Classmates on Adolescents’ Development of Prosocial Behavior

**Sensitivity Analysis: Calculation of Statistical Models Using Bootstrapping**

Table S1

*Multilevel Models Predicting T2 Prosocial Behavior Using Bayesian Significance Testing*

|  | **Model 1** | | | **Model 2** | | | |
| --- | --- | --- | --- | --- | --- | --- | --- |
|  | B | 95 % CI | ß | | B | 95 % CI | ß |
| Intercept | 1.37* | (1.25, 1.49) |  | | 1.36* | (1.24, 1.49) |  |
| Prosocial behavior (class level) | 0.44* | (0.39, 0.48) | 0.19 | | 0.44* | (0.39, 0.49) | 0.19 |
| Prosocial behavior (individual level) | 0.29* | (0.27, 0.30) | 0.30 | | 0.53* | (0.42, 0.64) | 0.56 |
| Gender | -0.07* | (-0.08, -0.06) | -0.09 | | -0.07* | (-0.08, -0.06) | -0.09 |
| Age | 0.02* | (0.01, 0.03) | .02 | | 0.02* | (0.005, 0.03) | 0.02 |
| Migration background | -0.05* | (-0.06, -0.03) | -0.05 | | -0.05* | (-0.06, -0.03) | -0.05 |
| Academic achievement | 0.06* | (0.04, 0.08) | 0.05 | | 0.06* | (0.04, 0.08) | 0.05 |
| Academic track | -0.04* | (-0.06, -0.01) | -0.04 | | -0.04* | (-0.06, -0.01) | -0.04 |
| Prosocial behavior (class level * individual level) |  |  |  | | -0.10* | (-0.14, -0.06) | -0.26 |

* *p* < .05

Table S2

*Multilevel Model Predicting T2 Prosocial Behavior by Gendered Prosocial Scores at T1 Using Bayesian Significance Testing*

| Predictor | B | 95% CI | | ß |
| --- | --- | --- | --- | --- |
| Intercept | 1.39* | (2.37, 2.48) | |  |
| Girls’ level of prosocial behavior (T1) | 0.22* | (0.19, 0.26) | | 0.12 |
| Boys’ level of prosocial behavior (T1) | 0.20* | (0.17, 0.24) | | 0.11 |
| Individual prosocial behavior (T1) | 0.54* | (0.28, 0.31) | | 0.54 |
| Gender | -0.08* | (-0.11, -0.09) | | -0.10 |
| Age | 0.02* | (0.004, 0.03) | | 0.02 |
| Migration background | -0.05* | (-0.06, -0.04) | | 0.05 |
| Academic achievement | 0.06* | (0.04, 0.08) | | 0.05 |
| Academic Track | -0.03* | (-0.06, -0.01) | | -0.04 |
| Girls’ level of prosocial behavior (T1) * Individual prosocial behavior (T1) | -0.04* | (-0.08, -0.004) | | -0.12 |
| Boys ’level of prosocial behavior (T1) * Individual prosocial behavior (T2) | -0.06* | (-0.10, -0.02) | | -0.14 |
| Girls’ level of prosocial behavior (T1) * Gender | -0.15* | (-0.18, -0.12) | | -0.47 |
| Boys’ level of prosocial behavior (T1) * Gender | 0.15* | (0.12, 0.18) | | 0.45 |
| Individual prosocial behavior (T1): Gender | -0.02* | (-0.01, 0.01) | | -0.02 |
| Girls’ level of prosocial behavior (T1) * Individual prosocial behavior (T1) * Gender | 0.01 | (-0.02, 0.05) | | 0.04 |
| Boys’ level of prosocial behavior (T1) * Individual prosocial behavior (T1) * Gender | -0.01 | (-0.04, 0.03) | | -0.02 |
| Observations | 16,707 |  | |  |
| Log Likelihood | -18,518.04 |  | |  |
| Akaike Inf. Crit. | 37,076.08 |  |  | |
| Bayesian Inf. Crit. | 37,230.55 |  |  | |

*Note*. The *N* is slightly lower than the total *N* of 16,893 because for a small number of classes that contained only male or female students, no gendered scores could be computed.

* *p* < .05.

Table S3

*Multilevel Models Predicting T2 Prosocial Behavior: Models without Covariates*

|  | **Model 1** | |  | **Model 2** | |  | | |  |  |
| --- | --- | --- | --- | --- | --- | --- | --- | --- | --- | --- |
|  | b | ß | *p* | b | ß | | | *p* | |  |
| Intercept | 1.16 | -0.01 | <.001 | 1.16 | -.002 | | <.001 | | |  |
| Prosocial behavior (class level) | 0.43 | 0.19 | <.001 | 0.43 | 0.19 | | <.001 | | |  |
| Prosocial behavior (individual level) | 0.30 | 0.32 | <.001 | 0.56 | 0.32 | | <.001 | | |  |
| Prosocial behavior (class level * individual level) |  |  |  | -0.10 | -0.04 | | <.001 | | |  |
| Observations | 17,783 | | | 17,783 | | | | | |  |
| Log Likelihood | -18,692.84 | | | -19,811.33 | | | | | |  |
| Akaike Inf. Crit. | 37,409.68 | | | 39,638.66 | | | | | |  |
| Bayesian Inf. Crit. | 37,502.50 | | | 39,700.95 | | | | | |  |

*Note*. The number of participants is higher in these analyses than the *N* of 16,893 for the main analyses due to missing cases when covariates are included.
